# Cyber Physical Systems Dependability Using CPS-IOT Monitoring

**DOI:** 10.3390/s21082761

**Published:** 2021-04-14

**Authors:** Antoine Bagula, Olasupo Ajayi, Hloniphani Maluleke

**Affiliations:** Department of Computer Science, University of the Western Cape, Bellville, Cape Town 7535, South Africa; ooajayi@uwc.ac.za (O.A.); 3219279@myuwc.ac.za (H.M.)

**Keywords:** collection tree protocols, cyber physical systems, dependability, epidemic modelling, fourth industrial revolution (4IR), internet-of-things (IoT)

## Abstract

Recently, vast investments have been made worldwide in developing Cyber-Physical Systems (CPS) as solutions to key socio-economic challenges. The Internet-of-Things (IoT) has also enjoyed widespread adoption, mostly for its ability to add “sensing” and “actuation” capabilities to existing CPS infrastructures. However, attention must be paid to the impact of IoT protocols on the dependability of CPS infrastructures. We address the issues of CPS dependability by using an epidemic model of the underlying dynamics within the CPS’ IoT subsystem (CPS-IoT) and an interference-aware routing reconfiguration. These help to efficiently monitor CPS infrastructure—avoiding routing oscillation, while improving its safety. The contributions of this paper are threefold. Firstly, a CPS orchestration model is proposed that relies upon: (i) Inbound surveillance and outbound actuation to improve dependability and (ii) a novel information diffusion model that uses epidemic states and diffusion sets to produce diffusion patterns across the CPS-IoT. Secondly, the proposed CPS orchestration model is numerically analysed to show its dependability for both sensitive and non-sensitive applications. Finally, a novel interference-aware clustering protocol called “INMP”, which enables network reconfiguration through migration of nodes across clusters, is proposed. It is then bench-marked against prominent IoT protocols to assess its impact on the dependability of the CPS.

## 1. Introduction

Cyber Physical Systems (CPS) are a new generation of systems that play a key role in interconnecting the physical and virtual worlds. Recently, vast investments have been made globally in the development of CPS, as they have been identified as key technologies that can boost the deployment of the Fourth Industrial Revolution (4IR). It is expected that CPS will pave the way for solutions to key economic and societal challenges, such as: Coping with an ageing population, improving issues of health and public safety, planning for mega-cities, achieving sustainability and globalisation, as well as proffering solutions to mobility challenges. Similarly, Internet-of-Things (IoT) principles are finding their way into next generation CPS, enabling extended interactive functionality between the physical and virtual environments through “sensing” and “actuation”.

By combining CPS and IoT, we gain the ability to infuse computing and communication capabilities into the dynamics of physical and engineered systems. This combination is expected to provide different ways of interacting and manipulating physical systems, through seamless network connectivity and refined user control over the actuation side. CPS are key 4IR components whose dependability is paramount to the operation of today’s critical infrastructures. As described in [1], the attributes of a dependable system include: Availability—readiness for correct service, reliability—continuity of correct service, safety—absence of catastrophic consequences on the user(s) and the environment, integrity—absence of improper system alteration, and maintainability—ease of maintenance (repair). CPS dependability is therefore among one of the most desirable characteristics of future CPS.

### 1.1. The Impacts of Emerging Trends on CPS-IoT

**Figure 1:**
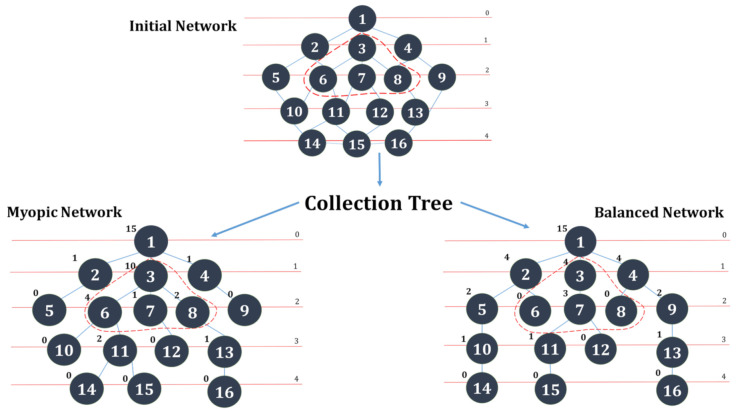
Relevance of efficient Cyber-Physical Systems-Internet-of-Things (CPS-IoT) monitoring.

CPS-IoT systems are often a combination of diverse subsystems, including numerous sensors from diverse manufacturers, varied gateways, and networking devices, as well as multiple actuation systems, all of which might run different protocols. The relevance of a dependable CPS-IoT system can therefore not be over-emphasised. However, serious considerations must be paid to the impacts of complex emerging IoT platforms and standards on CPS infrastructure. These impacts can be viewed from three dimensions, viz.:

1. The impact of complexity on CPS: By enabling information to be collected and communicated among everybody, everything, and from any where, CPS-IoT will usher in a new era where the cyber, physical, and human knowledge, as well as social activities are integrated into a unified platform. Though advantageous, the down side is that the CPS system might become very complex with heterogeneous components, multiple functionalities, rules, and feedback loops. These could lead to new kinds of risks and vulnerabilities. For instance, the failure of a single component could cascade into large-scale, system-wide failures as a result of the interconnection between the various components. The management of such complexity will require accurate modelling in order to guarantee the dependability of the CPS and the safety of the critical infrastructures it controls;
2. The impact of IoT standards on CPS: Current generation CPS are often managed by Orchestration Systems (OS), wherein physical processes are controlled by networks of sensors, actuators, and controllers. These networks are often built around static topologies with pre-planned routing and scheduling mechanisms mandated by standard wireless protocols such the WirelessHART [2]. Though these standards provide real-time guarantees for delay-sensitive applications, they do not consider performance-related tasks, such as management of resources at the physical layer, scheduling of nodes at the link layer, and/or end-to-end network layer routing of traffic. Next generation CPS will require performance-aware OS that are capable of dynamically interacting with the underlying IoT subsystem for sensing and actuation through IoT communication infrastructure as well as support lightweight CPS protocols. Finally, they would be capable of providing varied services to users while meeting the requirements of high throughput, high reliability, and energy efficiency yet operating within bounded communication delays;
3. The impact of information collection on CPS: The CPS of the future will be designed around a network infrastructure that interconnects islands of IoT networks, with information shared using a “m-to-1” network model from all the nodes connected to unique sink(s) rooted at the gateway(s). Collection Tree Protocols (CTPs) [3,4,5,6,7] are rapidly gaining ground in the IoT field and are predicted to dominate the CPS-IoT of the future. They are protocols that rely on spanning tree structures for information diffusion from sensor nodes to the sink through local structures referred to as diffusion sets in [8]. Sink nodes often serve as sources of beacon messages, broadcasted to guide routing updates. However, this raises the issue of a trade off between stability and safety. Frequent/excessive updates may result in routing oscillation, leading to the network wasting most of its resources in signalling instead of forwarding data to the sink. Conversely, infrequent updates may result in premature and/or delayed actuation and subsequent safety risks. This might result in sensitive portions of the IoT network entering an unsafe mode, damaging the controlled system, and/or lead to losses as illustrated in Figure 1.

As illustrated in Figure 1, two tree structures could be derived from the initial network, which are: (i) A myopic structure that may lead to some nodes being overloaded (such as node 3 carrying “10 children”) while other nodes are idle or underloaded (nodes 2 and 4 for example carrying only one child each) and (ii) a balanced network, wherein all nodes are comparatively evenly loaded.

When applied to a CPS such as a Cyber Physical Healthcare System or a Smart Building System, the collection tree structure may lead to the IoT network experiencing performance issues, especially when the node carrying a high number of descendants (e.g., nodes 3, 6, 7, or 8) is a critical node within a neo-natal unit of a hospital or a fire/emergency management unit of a smart building. This reveals the relevance of CPS-IoT dependability and its impact on systems that are controlled by it.

### 1.2. Contributions and Outline

The epidemic modelling of the CPS-IoT can help solve the issue of oscillation of the CPS-IoT during routing updates by (i) ensuring that routing updates are performed at the right moment when the stability of the network has moved far from its optimal point and (ii) providing a combination of epidemic states that may guarantee the safety of the CPS-IoT. Dependability is a key issue of future CPS that may be solved by redesigning current Collection Tree Protocols with enhanced safety features or proposing new dependability-aware protocols for CPS-IoT subsystems. This paper addresses the issues of dependability of CPS by building on (i) mathematical epidemiology to model the CPS-IoT as a dynamic system and (ii) interference-aware CPS-IoT reconfiguration to improve CPS dependability.

The contributions of this paper are threefold. Firstly, we propose a unified framework that builds upon a CPS orchestration model to efficiently manage and monitor the CPS. We then present a diffusion model for such a framework. While many epidemic models are based on a macro approach that consider only transitions between infectious groups, our work extends the state-of-the-art by including micro structures. These micro structures, called diffusion sets, reveal the propagation of information as defined by CTPs currently used in emerging IoT networking architectures. Secondly, a numerical analysis and validation of the efficacy of the proposed diffusion model with respect to network information flow and CPS dependability is presented. Finally, we propose a novel interference-aware clustering protocol that uses node migration between clusters as an efficient reconfiguration tool when the CPS-IoT has “drifted” from its safe operating state.

In this paper, CPS dependability is supported by (i) identifying a combination of epidemic state transitions that are more suitable for sensitive and non-sensitive applications and (ii) associating transition rates to these transition states and using them to re-optimise the CPS-IoT. Mathematical epidemiology has been used to model wireless sensor networks, mostly for mitigation against worm attacks. However, to the best of our knowledge, none of these models have addressed the issue of information dissemination based on the structures of the collection trees and diffusion sets as presented in this paper.

The rest of this paper is organised as follows. After this current section, related works are reviewed in Section 2. We then present the main components of the CPS orchestration model, and its underlying inner feedback loop and features in Section 3, thereafter, the epidemic dynamic model is proposed and its stability proven. The CPS monitoring model is described in Section 4, while in Section 5 the corresponding numerical results are presented. Section 6 then draws a conclusion and proposes some avenues for future research.

## 2. Related Work

Research work has been done to study issues related to different aspects of dependability of (i) Wireless Sensor Networks (WSN) as standalone systems or as components of the CPS and (ii) CPS as a whole. Some of these studies rely on mathematical epidemic models that describe population-level dynamics of infectious diseases.

### 2.1. Mathematical Epidemiology

The classic Susceptible-Infectious-Recovery (SIR) models were pioneered in 1927 [9]. They have been used to describe the interaction between individuals when a disease breaks out within a given population [10]. They are built around a compartmental structure where individuals are separated into compartments based on their infectious status. Each individual can only be in a single compartment at any given time but can transit between compartments with time. Since its inception, the original SIR-type framework has been adapted and used to solve problems in various domains including network problems [11,12,13,14]. More recently, it has been used in the prediction of the evolution of the Covid-19 pandemic [15,16,17,18] and the impact of vaccination to achieve herd immunity [19,20,21].

It has also been modified and extended into other models such as “E-SIRS”, “SIRS”, and “SIR-M”. Some of these extended models such as [11,12,22] are compartmental models, with their analysis being done using the basic reproduction number $R_{0}$, computed from a next generation matrix *K* [23,24]. In this paper, the basic SIR transition states have been modified to mimic a CPS-IoT network to achieve three objectives. (i) To analyse the evolution in time of the nodes of a CPS-IoT network, when considered as components of a population that has been compartmentalised in three epidemic states (Susceptible, Attacked (Infected), and Removed). (ii) Translating a combination of these states into dependability requirements. (iii) Re-configuring the nodes to restore the safety of the CPS-IoT whenever it operates outside its safety requirements. The SEIR, SIRS, and other models could also be adapted to mimic different behaviours and target other dependability requirements of the CPS-IoT, but these are beyond the scope of this work. These epidemic models can have tremendous impacts on the stability of a network when used to guide the routing updates and/or trigger network reconfiguration or re-optimisation when specific events occur.

### 2.2. Wireless Sensor Networks Dependability

In [25], a general framework using epidemic theory is presented for the analysis of vulnerability of broadcast protocols in WSN. The work assumes a form of vulnerability where an attacker can originate a piece of malicious code and use the broadcast protocol to gain control of sensor nodes or an important interrupt function. In [26], a microscopic mathematical model is proposed to describe the propagation dynamics of the sensor worm in Wireless Sensor and Actuator Networks (WSAN) in situations where actuator mobility causes sensor worms to spread faster. The model proposes a two-step Local Defending Strategy (LDS) with a mobile patcher designed for network recovery. In [27], a model formulated by differential equations is proposed to explain the process of worm propagation in WSNs. The model considers communication radius and area, nodal density, and the spreading dynamics of worms. While presenting similarities with our work in terms of epidemic modelling, the focus of these works [25,26,27] were on the monitoring and mitigation against attacks in a WSN. This paper goes beyond this and includes routing and rerouting processes to restore dependability in a CPS-IoT network.

### 2.3. Cyber Physical Systems Dependability

In [28], the authors proposed a cyber-physical management that includes a holistic controller that generates actuation signals for WSAN re-configuring for performance control. In [29], a methodology that includes an analytical model of CPS dependability behaviour and architecture is proposed. The model is applicable in environmental monitoring using WSN, multi-agent, and cloud computing technologies. In [30], an overview of different types of systems and the associated transition process from mechatronics to CPS and cloud-based (IoT) systems is presented. The paper also advocates for a multi-disciplinary CPS development process and presents challenges related to CPS design from various perspectives The content of paper [31] includes: A review of research and innovations on CPS reliability in Europe, procedures for evaluating the reliability of CPS sensors, and a co-simulation framework for real-time interaction between virtual and real sensors. An outlook of software and services supporting cyber physical systems with wireless sensor networks is presented in [32]. The focus of these papers [28,29,30,31] lies on CPS but none of them have addressed the dependability of CPS as presented in this paper.

### 2.4. Autonomic Computing

Autonomic computing provides the potential to transform the CPS of the future into a computing environment that can manage itself through a feedback loop using the MAPE structure initially proposed by IBM [33]. Using the MAPE structure, the CPS can be monitored through sensing, analysed through different modelling processes, and execute relevant mitigation processes through actuation when the CPS has moved away from its optimal operation. A decision-making framework for dynamically adjusting a dynamic IoT environment to changing requirements is proposed in [34]. The framework uses a combination of a finite-state machine model and a game theoretic decision-making method for extracting efficient strategies. The usability of the framework is demonstrated through a smart greenhouse-based use case. A Hierarchical fog-assisted Computing architecture (HiCH) is proposed in [35] for remote IoT-based patient monitoring systems with autonomous data management and processing performed at the edge of a network. Moving away from the conventional Observe-Decide-Act (ODA) control strategy [36], HiCH exploits and customises MAPE-K autonomic computing with an adaptive control loop that achieves monitoring in the sensor layer, analysis in the cloud layer, planning in the fog layer, and execution in the fog layer. A cloud-based middleware framework is proposed in [37] to provide a set of cloud services for self-adaptive IoT collaboration services in a cloud environment. In such an environment, domain-dependent components are separated from the layers that leverage existing middleware frameworks. Besides domain-dependent components and operations, the framework includes support for MAPE cycles on Virtual Machine and collaboration between multiple systems.

The literature surveyed above reveals a clear research gap in terms of traffic engineering techniques and models for CPS-IoT. It also points to the state-of-the-art in autonomic computing models for CPS-IoT dependability, which is gaining momentum with a number of papers applying self-configurable techniques to the IoT domain.

## 3. The CPS Dependability Model

**Figure 2:**
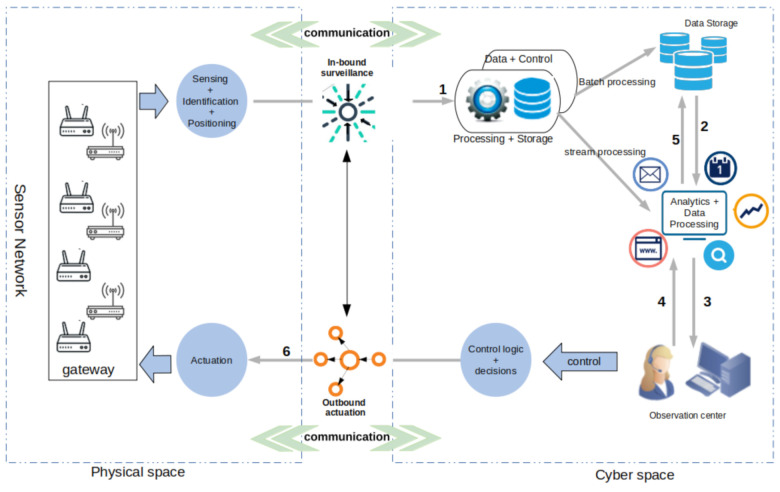
CPS orchestration model.

The CPS considered in this paper is built around an enhanced network orchestration model with inner feedback loop that reveals the different components and processes involved in its management. As depicted by Figure 2, the model shows how (i) inbound surveillance is performed on the network information collected from the IoT subsystems and (ii) outbound actuation is used to control the operation of the CPS-IoT from a safety perspective.

### 3.1. The CPS Orchestration Model

In Figure 2 an inner feedback loop reveals the interactions between the cyber and physical spaces and how the CPS is monitored through an inbound surveillance and an outbound actuation process. The figure also reveals: (1) A physical-to-cyber flow of information originating in the physical space and translated into services at higher levels in the cyber space and (2) a cyber-to-physical flow of information, initiated by the processing of information in the cyber space to achieve actuation, both within the sensor network and on physical objects in the physical space. In essence, information flowing from the physical to the cyber space includes: “Sensor data” obtained from the sensing of physical objects and “sensor network monitoring data”, which are extracted from sensor network surveillance. Information flowing from the cyber to the physical space on the other hand contain “control signals” for actuating physical devices within the environment and “control messages” required for re-optimisation and reconfiguration of the sensor network. In the proposed CPS management model, the key processes are sequentially iterated in a loop as follows:

Sensing: This is initiated in the physical space to sense the environment for further processing in the cyber space. It generates “sensing data”, which are routed over paths created by CTPs. These sensing data are used to analyse the diffusion of information in the CPS-IoT subsystem, for the purpose of monitoring the performance of the subsystem and and anomaly detection.

Communication: The communication process is initiated at the physical space where IoT protocols such as LoRa, ZigBee, and BLE are used to move data from sensors to sink nodes and finally to gateways at the edge layer. Communication processes can also extend to the cyber space where other protocols are used to move data from the gateways to the fog layer for further processing.

Inbound surveillance: This is the first process initiated in the cyber space and is used to assess the integrity and performance of the CPS’s IoT subsystem as well as detect anomalies before and during data processing.

Data + Control: Filters data and control information and stores the information into databases for batch or stream processing. In batch processing, data are stored in databases for a longer period of time while streamed information are processed in real time to produce data analytics for decision making.

Data processing: Here various analytics and simulations are applied to the data (both batch and stream) to support decision systems.

Observation center: The observation center is an entity of the CPS management infrastructure where the results are observed by the operation specialists for decision-making purposes (storage or actuation).

Outbound actuation: The outbound actuation is the last process implemented at the cyber space. It involves adjusting the operating parameters of the IoT subsystem based on the inbound surveillance to restore the CPS’s optimal settings whenever necessary.

Actuation: Initiated in the physical space to either restore the network to its optimal operational level or perform actuation on physical objects in the environment as a result of the data analytics or decision(s) made at the observation center.

As described above, the CPS orchestration model includes an adaptive control loop similar to the MAPE-K structure [33,34,35,36,37] where (i) “Monitoring” is achieved through the sensing process, (ii) the “Analysis” is done by the inbound surveillance, (iii) “Planing” is performed through the Data processing process, (iv) the “Execute” is achieved by the outbound actuation process, and (v) the “Knowledge” acquired in the observation center is used to support decision making.

### 3.2. Epidemic Modelling of the CPS-IoT

**Figure 3:**
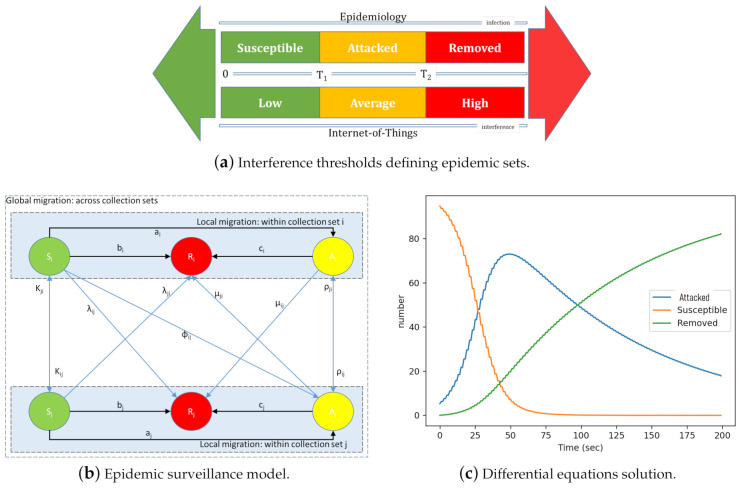
The epidemic modelling.

As illustrated in Figure 3a, the states of the nodes of the CPS-IoT can be mapped into epidemic states when considering: (i) The risks and levels of congestion associated with the network collection tree topology and connectivity and (ii) the energy that can be drawn from nodes as a result of such topology and connectivity. As IoT networking uses CTPs, the number of “children” carried by a node (node interference), can be translated into an epidemic state expressing the level of contamination of the node. In this paper, two interference thresholds, $T_{1}$ and $T_{2}$, have been used to map epidemic states into IoT networking states. Building around the SIR epidemic model, this paper considers the SAR model that uses three epidemic states referred to as Susceptible (safe), Attacked, and Removed statuses. These states are respectively the loose equivalent of the Susceptible, Infected, and Removed states of the SIR model. We define the considered states as follows:

Susceptible nodes: Are the least or non-interfering nodes in a network. Their total number is denoted by *S*. Each susceptible node *n* is assumed to have weight (level of interference) less than the threshold $T_{1}$.

Attacked nodes: Are highly interfering but still operational nodes. The total number of infected nodes in a network is denoted by *A*. An infected node is assumed to have weight less than the threshold $T_{2}$ but at least equal to the threshold $T_{1}$.

Removed nodes: Are nodes that are no longer operational as a result of high levels of interference between themselves. These nodes are also referred to as depleted and their total number is denoted by *R*. A node is considered to be removed if its interference is at least equal to the threshold $T_{2}$.

Note that the transitions from Susceptible to Attacked (S-to-A) and from Attacked to Removed (A-to-R), may determine the IoT’s safety level. From a CPS-IoT safety perspective, high availability translates to a “healthy” or “uninfected” state, while reliability translates to the ability of a node to accurately and safely carry out its function(s). An analysis of dependability based on the epidemic model proposed in this paper may thus lead to four potential cases depicted in Table 1. Higher migration rates from a state to another are represented by binary values “1” while lower migration rates are represented by binary numbers “0”.

- Higher transition rates from a susceptible to an attacked state is an indication of an IoT network with low availability. This is because nodes will move faster from the susceptible (safe) state to the attacked state;
- Lower transition rates from the susceptible to attacked state are an indication of an IoT network with higher availability; that is nodes stay longer in the susceptible state;
- Higher transition rates from attacked to depleted state are an indication of a more reliable IoT network. This is because the infected (attacked) nodes are quickly removed to minimise potential risks to the IoT network;
- Lower transition rates from an attached to removed (depleted) state are an indication of a less reliable IoT network. This is because nodes stay longer in an attacked state, hence increasing the potential risks to the CPS and its IoT network sub-system.

Note that as reported in Table 1, the best performance in terms of dependability (safety) is achieved in Case 4. This applies to sensitive applications with hard real-time constraints, which should be deployed with a low migration rate from susceptible to attacked status $a_{i}$ and a high migration rate from attacked to removed status $b_{i}$. Such applications will require higher surveillance in terms of anomaly detection and quick response/actuation. For instance, this can be implemented using interference-aware data collection algorithms (such as Least Interference Beaconing Protocol (LIBP) [38]) that can prevent IoT nodes from moving into the attacked state. Similarly, Table 1 reveals that the average performance in terms of dependability is achieved in Case 2, when the IoT nodes stay longer in the susceptible and attacked states before being removed. Case 1 and Case 3 depict low dependability situations with the worst scenario being Case 3, where the nodes spend less time in both the susceptible and attacked states. Case 2 applies to non-sensitive applications with soft real-time constraints that should be deployed with low migration rates from susceptible to attacked status $a_{i}$ and from attacked to removed state $b_{i}$. Such applications will require less surveillance in terms of anomaly detection and slower response/actuation by implementing cost aware data collection algorithms such as Routing Protocol for Low-Power and Lossy Networks (RPL) [6]. The analysis of the dependability/safety cases when triggered by transition rates adjustments is presented in Section 5. The comparison between the CTPs in terms of epidemic status levels and their relevance in CPS dependability will also be evaluated in Section 5.

In modelling these transitions epidemically, Figure 3b presents a finite state machine of the epidemic model upon which the dependability of the CPS relies upon. It depicts a network that has been partitioned into a number of diffusion sets, with nodes in the same diffusion set assumed to be infectiously similar to each others. The figure reveals the states of the diffusion sets and for each state its associated transitions as well as the actions that trigger such state transitions. Susceptible nodes in the diffusion set $I_{ı}$ may be attacked at the rate $a_{i}$, while the attacked nodes from $I_{ı}$ are removed at rate $c_{i}$. Susceptible nodes in the diffusion set $I_{ı}$ may experience high interference, enough to move them directly to the removed status without transiting through the attacked status. On the other hand, removed nodes my cause some of the susceptible or attacked nodes to leave the network because of the destruction of connection links. We consider $b_{i}$ to be the rate at which susceptible nodes in $I_{ı}$ are removed. Susceptible nodes from diffusion set $I_{ı}$ migrate to diffusion set $I_{j}$ with rate $\lambda_{ij}$, and attacked (or infected) nodes in $I_{ı}$ migrate to $I_{j}$ at a rate of $\rho_{ij}$. In contrast to the classic SIR epidemic model, our model allows susceptible nodes to move directly into the removed state. This reflects the case of nodes that are removed from the system for reasons other than the infection/attack, such as component failure or malfunction. The difference in equations resulting from these assumptions will be presented as constraints of the CPS monitoring model proposed in Section 4. Using the Euler method, this system of differential equations can be solved to produce three graphs representing the evolution of the populations *S*(*t*), *A*(*t*), and *R*(*t*) over time as presented in Figure 3c.

With regards to CPS safety, these cases may be translated into an objective optimisation problem, consisting of maximising or minimising the surface below the curves *S*(*t*), *A*(*t*), and *R*(*t*). This can be expressed by the integral of these curves over time and defined as:

(1) $$f\left(t\right)=\max\int_{0}^{T}S_{ı}\left(t\right)\pm \int_{0}^{T}A_{ı}\left(t\right)\mp \int_{0}^{T}R_{ı}\left(t\right)$$

where *T* is the study period of the epidemic model and ± express a maximisation or minimisation of the surface below the epidemic curve in relation to Table 1.

## 4. The CPS-IoT Monitoring Model

The diffusion sets are local data structures that reveal the impact of “data transportation” on IoT network nodes. Their structure can reveal which nodes will be more burdened compared to others as well as the potential risk of transferring interference across nodes. As stated earlier, in our diffusion model, nodes are grouped into diffusion sets with nodes in the same diffusion set assumed to be infectiously similar, while those in different sets are dissimilar. This section presents an analytical formulation of the inbound surveillance and outbound actuation models and how these models are analytically solved.

### 4.1. The CPS Monitoring Problem

In our model, each diffusion set $D_{ı}$ comprises of subsets of nodes defined by $D_{ı}=\left\{S_{ı},A_{ı},R_{ı}\right\}$. The subset $S_{ı}$ of **susceptible nodes**, which are working and uninfected nodes, is of a size denoted by $S_{i}$, while the subset of **attacked nodes** is of size $A_{ı}$. Finally the subset of **removed nodes** is of size $R_{i}$. When the diffusion set $D_{ı}$ includes both the attacked and removed sets that are non empty ($A_{i}\neq 0$ or $R_{i}\neq 0$), it is said to be an **infected set**.

The inbound surveillance problem consists of finding for each diffusion set $D_{ı}$ its evolution function over time $I_{ı}\left(t\right)=\left\{S_{i}\left(t\right),A_{i}\left(t\right),R_{i}\left(t\right)\right\}$, while taking into account the state transitions (Figure 3b) and the difference equations behind such transitions. On the other hand, the outbound actuation problem consists of finding the right collection tree protocol and applying that protocol to restore CPS dependability when its underlying CPS-IoT subcomponent has moved away from its optimal/safe operating conditions. The CPS monitoring problem is defined formally as follows:

(2) $$\left\{\begin{array}{cc} \max\int_{0}^{T}S_{ı}\left(t\right)\pm \int_{0}^{T}A_{ı}\left(t\right)\mp \int_{0}^{T}R_{ı}\left(t\right)) & \\ \mathrm{subject}\\ \mathrm{to}\left\{\begin{array}{cc} \forall x\in S;\to w\left(x\right)<T_{1} & \left(2-1\right)\\ \forall x\in A;\to T_{1}\leq w\left(x\right)<T_{2} & \left(2-2\right)\\ \forall x\in R;\to w\left(x\right)\geq T_{2} & \left(2-3\right)\\ S_{i}^{{}^{\prime }}=-a_{i}S_{i}+\sum_{j\neq i}\lambda_{ji}S_{j}-\sum_{j\neq i}\lambda_{ij}S_{i}-b_{i}S_{i} & \left(2-4\right)\\ A_{i}^{{}^{\prime }}=a_{i}S_{i}+\sum_{j\neq i}\rho_{ji}A_{j}-\sum_{j\neq i}\rho_{ij}A_{i}-c_{i}A_{i} & \left(2-5\right)\\ R_{i}^{{}^{\prime }}=b_{i}S_{i}+c_{i}A_{i} & \left(2-6\right). \end{array}\right. \end{array}\right.$$

$\forall S_{ı},A_{j},R_{j}\in E$ where $a_{i}$ is the transmission rate between susceptible and attacked nodes, $b_{i}$ is the migration rate from susceptible diffusion set $S_{ı}$ to attacked diffusion set $A_{j}$, while $c_{i}$ is the migration rate from attacked diffusion set $A_{ı}$ to removed diffusion set $R_{j}$. Note that the diffusion model does not express any dependability constraints. It only expresses a set finding function and how it is mapped into: (i) A set finding problem expressed by the routing objective (1). Equations (2-1)–(2-3) express the network partitioning into epidemic states: Susceptible: S, attacked: A, and removed: R. Equations (2-4)–(2-6) are the set of differential equations representing the state diagram in Figure 3b showing how the information and related interference is diffused from one set to another and reveal the impact of moving nodes from the susceptible to infected states (attacked or removed) on the network. These constraints reveal (i) that the migration rate from susceptible to attacked reduces with an increase in the threshold $T_{1}$ and vice versa, (ii) similarly, the migration rate from attacked to removed decreases with an increase in threshold $T_{2}$, and (iii) the network impact depends on both thresholds $T_{1}$ and $T_{2}$. The parameter $a_{i}$ directly depends on the number of susceptible nodes $S_{i}$ and the attacked ones $A_{i}$. It therefore makes sense to relate $a_{i}$ with two other measures: (i) The susceptibility rate of each node in the diffusion set $D_{ı}$, denoted by $\beta_{i}$ and (ii) the infectiousness rate of nodes in the infected diffusion set $I_{ı}$, denoted by $\gamma_{i}$.

Conversely, the structure of a diffusion set clearly influences the attack ability, as the effects of infections vary across different diffusion sets. We use the parameter $\eta_{i}$ as a measure of the impact on the network structure when a node gets infected (attacked or removed). Therefore, $a_{i}$ can be computed using the following formula:

(3) $$a_{i}=\beta_{i}\gamma_{i}\eta_{i}\frac{A_{i}}{N}$$

where $\frac{A_{i}}{N}$ denotes the fraction of infected nodes in diffusion set $I_{ı}$, and $\eta_{i}$ is a parameter revealing the impact of the diffusion on the network if a susceptible node in a diffusion set $S_{ı}$ becomes infected (attacked or removed).

### 4.2. CPS Monitoring Implementation Model

**Figure 4:**
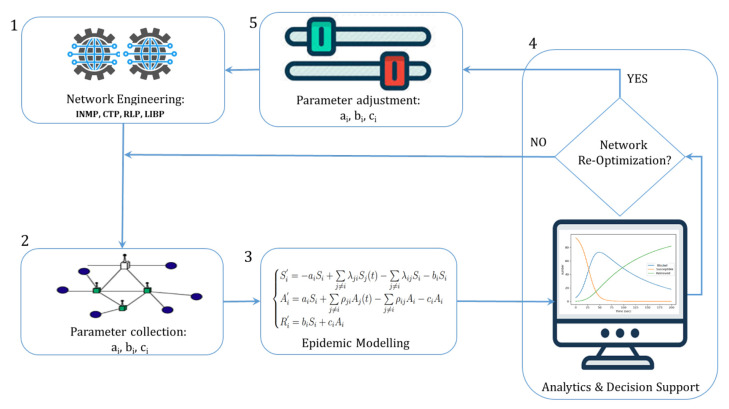
CPS monitoring model.

An implementation of the CPS monitoring model is depicted by Figure 4, which depicts the different processes involved in the solution to the CSP monitoring problem. The figure reveals a network monitoring loop that contains: (1) A network engineering module that computes the diffusion sets and uses RPL, LIBP, or Collection Tree Protocol (CTP) protocols to build collection trees; (2) collection of the transition rates parameters in a live IoT network; (3) an epidemic modelling of the IoT network as a dynamic system; (4) performance analysis of the dynamic system using analytics and visualisation of the epidemic curves to detect performance degradation; and (5) network recovery by applying the right collection tree protocol to re-optimise the CPS-IoT when it has moved far from its safe state of operation.

### 4.3. Algorithmic Solutions

The solution to the inbound surveillance problem consists of applying the Euler method to solve the systems of Equations (2-4)–(2-6). Actuation on the other hand is achieved through: (i) Visualisation and analysis of the epidemic curves resulting from the Euler solution, (ii) either adjusting the parameters $b_{ı}$ and $c_{ı}$ to restore the CPS-IoT to its safe operating status and/or associating this parameters’ adjustment to a network re-optimisation process performed in the routing process, or (iii) solely performing a global network re-optimisation using an efficient (different) protocol to restore the CPS-IoT. While the study of a network re-optimisation process using parameter optimisation is beyond the scope of this study, a comparison between interference-aware and interference-myopic algorithms in terms of the epidemic states reached by common CTPs is presented in Section 5 to reveal the relative performance of these protocols in terms of CPS safety. Furthermore, a novel interference-aware clustering protocol called Interference-aware clustering with Node Migration (INMP) is proposed to restore the safety of the CPS.

### 4.4. Interference-Aware Clustering with Node Migration Protocol (INMP)

Interference-aware clustering with node migration (INMP) is an extension of the IoT multi-sink protocol in [4] and is built around the concept of node migration/sharing between clusters to improve the CPS-IoT performance (and safety).

#### 4.4.1. The Node Migration Concept

**Figure 5:**
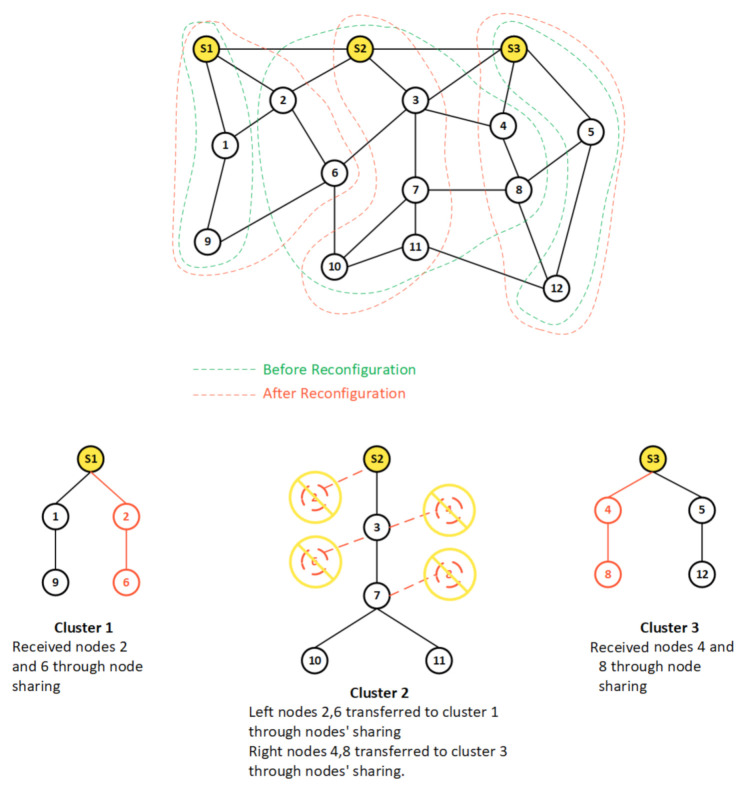
Node-sharing model.

Figure 5 illustrates the node migration paradigm for a network represented by a graph of 15 nodes including three sink nodes $S_{1}$, $S_{2}$, and $S_{3}$. The figure depicts a scenario where the three initial clusters have 3, 8, and 3 nodes respectively, leading to an unbalanced CPS-IoT network configuration with cluster 2 overburdened while the others are lightly loaded. In such a scenario, much higher energy consumption will be experienced at the sink node $S_{2}$ compared to $S_{1}$ and $S_{3}$. The figure also reveals how through node migration, clusters 1 and 3 each received two additional nodes from the overburdened cluster 2, thus resulting in a more balanced CPS-IoT network environment. This migration of nodes can be potentially beneficial in terms of energy consumption and network infrastructure safety. In the figure, the initial network graph is shown on the top, with the green dashes showing its partitioning into clusters before node migration, while the red dashes reveal the partitioning after migration. The collection trees related to the different clusters are represented in the lower part of the figure with the broken red lines revealing nodes that have been discarded from the initial tree while the full red lines reveal nodes that have been added through node migration.

Reorganising clusters for a load balancing purpose is an efficient strategy for self-organising systems. The works presented in [39,40] reorganise clusters for load-balancing with a focus on (i) balancing the processing load among fog nodes in [39] by having compute nodes located in the corresponding fogs measuring the network proximity to each other and self-organising into a hierarchical or a flat structure accordingly and (ii) providing resilient distributed decision-making in large-scale situated systems by devising a process of interconnecting edge devices into teams and applying a decentralised coordination pattern for partitioned integration and coordination of these devices, which continuously adapt to context change as proposed in [40]. The work presented in this paper applies a clusters’ reorganisation model but with a focus on the CPS-IoT reconfiguration using a balanced cluster membership based on node migration.

#### 4.4.2. The INMP Protocol

The INMP protocol is based on the the following key features:

- The CPS-IoT is subdivided into n clusters, each cluster i∈n being of size k(i);
- Each cluster is organised into a m-to-1 model using a collection tree structure with the root being a single sink node S(i);
- Clusters in an INMP map to collection sets;
- In contrast to the multi-sink structure proposed in [4], node migration can be performed between clusters to restore the CPS-IoT safety and this is triggered by routing updates;
- The routing updates are triggered by either: (i) A beaconing message resulting from visualising the epidemic model or computations using the epidemic curves or (ii) monitoring of the diffusion sets, which reveals that nodes in the sensitive diffusion sets are rapidly moving far from their prescribed epidemic states or that most of the nodes are in an infected epidemic state. This step is carried out at the observation centre (steps 2 to 5 in Figure 2 or step 4 in Figure 4);
- The number of nodes moving from one cluster to another may depend on many parameters including: (i) The difference in sizes of clusters with the expectation of balancing the load between clusters; (ii) the average and maximum energy consumed by the sink nodes of the clusters; and (iii) the network epidemic state, which reveals the level of attack on the network;
- The inbound surveillance is a two-steps process that starts with the physical environment where the CPS-IoT network performance is evaluated to reveal if some clusters have sink nodes with energy levels above the cluster average. This is used in a second step to trigger the computation of the SAR system of ordinary differential equations in the cyber space to reveal the epidemic evolution in the diffusion sets;
- The outbound actuation is also a two-steps process that starts with a simulation of the optimal/efficient configuration of the CPS-IoT clusters, which is followed by the transfer of the new configuration into the CPS-IoT network in the physical space through parameter adjustments.

**Figure 6:**
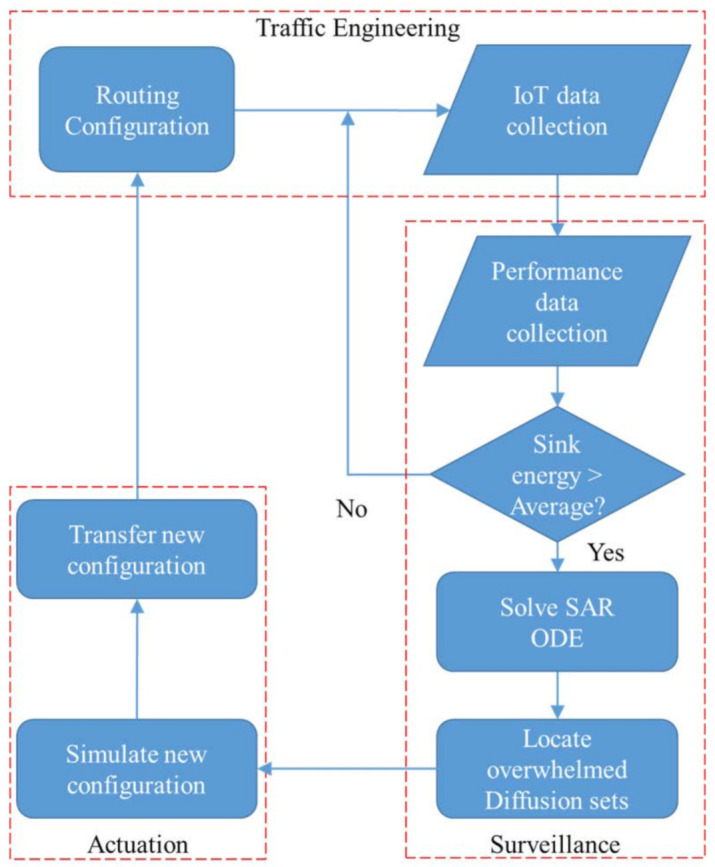
The Interference aware clustering with Node Migration Algorithm (INMA) flowchart.

The INMP protocol is an implementation of the Interference aware clustering with Node Migration Algorithm (INMA) described in Figure 6.

Note that as presented in this paper, the INMA algorithm contains a loop that includes some steps of the MAPE-K control loop, introduced by IBM [33] and adopted in different works on autonomic computing and self-organising networking [33,34,35]. It monitors and collects details such as topology information, metrics (e.g., offered capacity and throughput), configuration property settings, etc. from managed resources, then performs complex data analysis and reasoning on the symptoms (information) collected.

## 5. Performance Evaluation

In this section, reports of the numerical analysis of the diffusion model in relation to the diffusion and epidemic sets are presented.

### 5.1. The Performance Parameters

We conducted a number of experiments to analyse the performance of the compartmental epidemic model with the objective of revealing how they might be used to monitor the CPS’s IoT subsystems. The first results presented in this section are related to the difference equations expressed by (2-4)–(2-6) and solved using the Euler method described in [41]. The first set of experiments were conducted to evaluate the efficiency of the epidemic model in mimicking information diffusion in IoT networks. The second set were designed to find the impact of CTPs on the CPS-IoT subsystem. For the first set of experiments, the numerical computation and corresponding graphs were done using Python in Jupyter notebook IDE, with Cooja running on Contiki OS [42] to produce the results of the second experiment. Table 2 shows the initial conditions of the considered network and its performance parameters.

For this experiment a setup consisting of 10 {S,a,b…i} nodes was used. One node, assigned the sink node *S*, was connected to a gateway and powered externally, while the other nodes were battery powered and forwarded all data to *S*. We considered two key performance metrics:

(i) Performance patterns $\left[S_{i},A_{i},R_{i}\right]$ are functions that show the evolution of routing process over time. They reveal how nodes move from states to states and across diffusion sets. (ii) State-to-state migration expresses the rate by which nodes change states in the order: S, A, and R. It includes two parameters: The safe to attacked migration expressed by s−2−a and the attacked to removed migration expressed by a−2−r.

### 5.2. Inbound Surveillance

**Figure 7:**
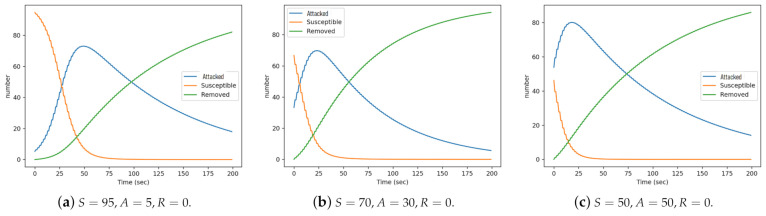
Inbound surveillance scenarios.

We conducted a number of simulations to determine a performance pattern of the diffusion sets under varied conditions. To study the effect of inbound surveillance, we considered four experimental scenarios corresponding to four diffusion sets. In the first $I_{1}$ we set {S=95,A=5,R=0}, this depicts an ideal initial condition where most of the nodes are in the safe state and batteries are mostly fully charged. For $I_{2}$, $I_{3}$, and $I_{4}$, we set {S=70,A=30,R=0}, {S=60,A=40,R=0}, and {S=50,A=50,R=0} respectively representing a system with 70%, 60%, and 50% of their nodes in safe states. The settings of $I_{2}$ and $I_{3}$ were similar, hence only that of $I_{2}$ is reported for the purpose of brevity. Figure 7 depicting three of these four scenarios reveal how inbound surveillance can be achieved by tracking the evolution in time of the epidemic curves. Scenario $I_{1}$ is depicted by Figure 7a while Figure 7b illustrates scenario $I_{2}$. Scenario $I_{4}$ is depicted by Figure 7c. Different other scenarios depicted by different epidemic curves can emerge from the inbound surveillance to reveal different network conditions when monitoring the CPS-IoT.

Susceptible states: In all three diffusion sets, a ’birth-decrease’ pattern was observed where the number of susceptible nodes gradually reduces as nodes transit from the susceptible to infected (attacked or removed). In Figure 7a, it took about 60 s for all susceptible nodes to be transited to other states, while it took about 35 s in scenario 2 and only 20 s in the third scenario.

Attacked states: Across all scenarios, a ‘birth-growth-decrease’ was observed at the attacked state. As the interference level on susceptible nodes increased (as a result of accepting more child nodes), their numbers reduced until they reached the threshold ($T_{1}$), above which they migrated to the attacked state. In scenario 1, the number of susceptible and attacked nodes were equal (50 each) after about 20 s, with the attack nodes peaking at 50 s. It is important to note that at its peak, there were only about 70 nodes in the attacked state. This is because some nodes had migrated directly to the removed state.

Removed states: A ‘birth-growth-plateau’ pattern was observed in all scenarios. Here the number of removed (battery depleted) nodes initially rose quickly until it equaled the number of attacked, after which the growth rate reduced slightly. In scenarios 1 and 3, not all nodes were removed, as some nodes remained in the attacked state at the end of the experiment.

### 5.3. Outbound Actuation

**Figure 8:**
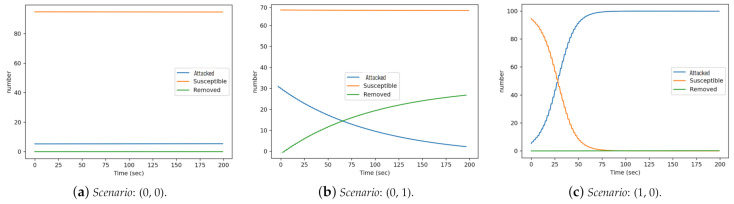
Outbound actuation scenarios: (s−2−a,a−2−r).

In this section, we study the effect of changing migration rates by mostly considering the first scenario, where the number of nodes were set to {S=95,A=5,R=0}. To achieve this, we changed the migration rates and evaluated the impact of such adjustment on the dependability of the CPS-IoT subsystem. These changes were based on Table 1 and with respect to: Availability—when there is the need to keep the nodes in the susceptible states for longer duration before migrating to the infected states and reliability—where the focus is to avoid nodes staying too long in the attacked state but instead migrate quickly to the removed state, in order to avoid compromising the system’s overall integrity. The experimental results are reported in Figure 8 which depicts three scenarios revealing how outbound actuation will impact the CPS-IoT.

#### 5.3.1. s−2−a=0,a−2−r=0

Setting s−2−a=0 translates to nodes staying longer in the susceptible states, thus being highly available while a−2−r=0 implies low reliability, as nodes stay longer in the attacked state. By setting both values to zero, migrations across states were cancelled out. These implies that nodes remained in their initial states throughout the duration of the experiment. The result is as depicted in Figure 8a where all the lines remained flat and unchanged throughout with no migration across states.

#### 5.3.2. s−2−a=0,a−2−r=1

For this, the migration rate from susceptible to attacked was set to 0, in a bid to keep nodes longer in the susceptible state while encouraging faster migration to the removed state. This translates to high availability and high reliability. In order to better show the impact of migration from attacked to removed, we used scenario 2: {S=70,A=30,R=0} as there were more nodes in the attacked state at the beginning of the experiment. Figure 8b, showed that the number of susceptible nodes remained unchanged, this is expected as the migration rate s−2−a was set to zero. Compared to Figure 7b, the attack curve shows a ‘birth-decrease’ instead of a ‘birth-growth-decrease’. There was no ‘growth’ in the number of attacked nodes because there was no migration from susceptible to the attacked state, only from the attacked to the removed. This also accounts for the inverse but equal growth rate observed with the removed curve.

#### 5.3.3. s−2−a=1,a−2−r=0

Here the migration rates from susceptible to attacked was set to 1, while the migration from attacked to removed was set to 0. This was done to target low availability and low dependability, where the nodes move quickly out of the susceptible state but stay longer in the attacked state. Figure 8c, shows that the removed curved remained flat as there was no migration to the removed state (since a−2−r was set to 0). The susceptible curve dropped rapidly, reaching 0 at about 75 s, while the attacked curve grew rapidly from 5 nodes and plateauing at 100 nodes after 75 s. All the nodes ended up in the attacked state state at the end of the experiment.

#### 5.3.4. s−2−a=1,a−2−r=1

When the migration rates were both set to 1 that is quick migration across states. This translates to low availability and high reliability. Obtained results were similar to those in Figure 7a, with nodes rapidly migrating out of the susceptible state to other states. Similarly, the attacked curve grew rapidly as nodes migrated from the susceptible into the attacked state until a peak was reach. It then rapidly dropped towards 0, as nodes migrated out of the attacked to the removed state.

### 5.4. The Impact of CTP, RPL, and LIBP on CPS Dependability

As suggested earlier, the existing CTPs may have a varied impact on CPS dependability of sensitive or non-sensitive applications. While non-sensitive applications can support an extended operation with nodes in the attacked status, sensitive applications will rather favour an extended operation with nodes in the susceptible states and tolerate only a minimised operation with nodes in the attacked state to avoid putting the CPS safety at risk. The impact of such protocols on the dependability of the CPS has been evaluated in this section by conducting experiments to evaluate the epidemic status levels reached by IoT nodes when data collection is guided by three CTPs (CTP, RPL, and LIBP) under the similar threshold values $T_{1}$ and $T_{2}$. The experiments were conducted on an emulated Tmote sky mote on the Contiki platform running on Cooja. UDGM (Distance Loss) was considered as the radio medium of choice. The default implementations of CTP [5] and RPL [6] protocols on Contiki were used, while LIBP was forked from the Contiki implementation of the CTP protocol. This was done by disabling the trickle algorithm and modifying the code to meet the requirements expressed by the compartmental routing problem formulation. In our experiments, the RPL protocol was run as two experimental instances using various Objective Functions (OF) referred to as RPL-0 or RPL-ETX. For this work, Cooja parameters were set similar to those of LIBP [4,38]. The experiment runtime was based on the following key features:

- Upon starting up, 2 min were given to allow each network “settle”, thereafter the network ran for 8 min, giving a total simulation runtime of 10 min;
- Each node periodically sent a packet containing the string “Hello from node” as its packet data (payload). The payloads were sent at a 30 s time interval over the 8 min duration. This resulted in each node sending 16 packets data to be collected by the sink;
- For the various experiments, Cooja’s existing profiling tools were used. For time sensitive experiments, both the simulation timers and node real-time timers were used to ensure accuracy;
- To distinguish between control and data plane traffic, packets were tagged. Counters were updated when specific packets types were received;
- All experimental results are presented with epidemic colours—blue, red, and green, linked to the amount of nodes in a specific epidemic state (susceptible, attacked, or removed). This was applied to all three routing protocols (LIBP, CTP, and RPL) tested to reveal their suitability for the safety of the CPS. This helps putting these results into the context of the CPS dependability under study.

#### 5.4.1. Energy Profile

**Figure 9:**
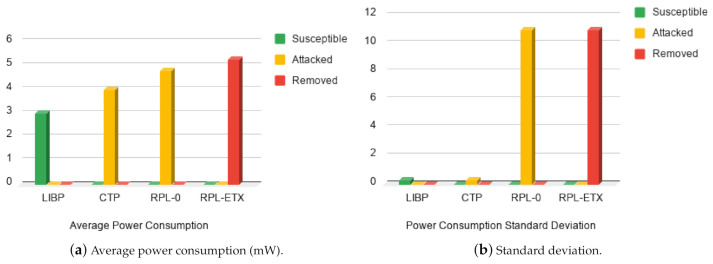
Energy profile: Average power consumption.

A comparison of the average power consumption of each node for the various protocols is shown in Figure 9a. The graph shows that RPL is on average significantly more power hungry than CTP and LIBP as most of its nodes were in the removed state with battery depleted after the simulation period. This could be due to the fact that the in RPL, sink node’s is always on and that RPL is built upon a heavyweight communication protocol. The standard deviation of power consumption describes how well distributed the energy consumption within a topology is. It is a metric that gives insights to the running time of a network and how long it can run before its nodes’ batteries are depleted. Low energy usage and low standard deviation shows that the protocol is energy efficient both in its distribution and implementation. Figure 9b shows that RPL-0 has the highest deviation for attacked nodes, while removed nodes have the highest deviation for RPL-ETX.

#### 5.4.2. Routing Profile

LIBP’s main routing metric consists of the interference defined by the number of supporting children per node. It is also an important parameter that defines not only the reliability of the routing topology resulting from a routing protocol but also the diffusion process. The path length is a performance parameter that reveals how efficiently a sensor network has been engineered.

**Figure 10:**
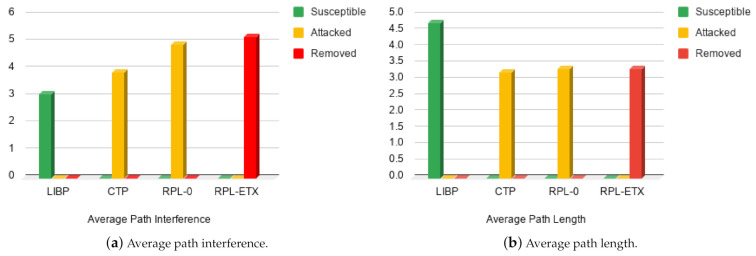
Routing profile: Interference and path length.

Figure 10a shows the average path interference in terms of the average number of children supported per node. It was obtained by averaging the number of times each node referenced its parent. Smaller values are desirable and indicate balanced load distribution. A balanced load implies a fair and efficient energy distribution in the network and longer lifetime for the entire sensor networks, as all the nodes would most likely have equal battery utilisation. On the other hand, higher interference values are undesirable, as they are indications of congestion that may lead to a higher rate of packet loss across the network. The results presented in the figure show that LIBP does better in this experiment compared to CTP and RPL.

Depending on the application, a high average path length (leading to a deeper routing tree) may be desirable for better energy distribution. Conversely, a lower average path length (shallow routing tree) can result in lower latency between leaf nodes and sink nodes. The average path lengths depicted in Figure 10b were obtained by computing a Time To Live (TTL)-like attribute within the protocol’s control plane packets. LIBP and RPL use TTL, while CTP uses “Time has lived” (which is TTL_MAX−TTL). Once the number of hops was obtained they were averaged to give an average path length metric for each protocol. These results show that on the average, LIBP expands its routing tree by 2 hops compared to the other protocols.

#### 5.4.3. Epidemic Profile

**Figure 11:**
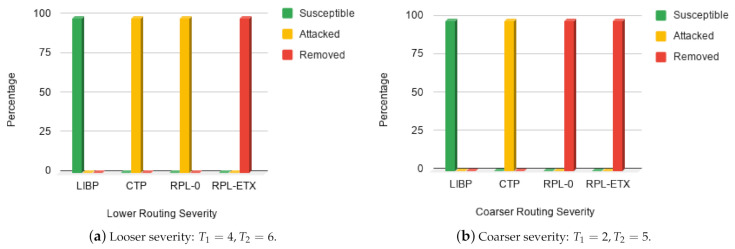
Epidemic profile: Interference and path length.

The interference thresholds determine the severity of the routing process in terms of migration of nodes from one epidemic group to another. Looser severity might lead to more IoT nodes staying longer in the susceptible state, while coarser severity may move more nodes into the removed states. The results depicted by Figure 11 represent the average states of the network nodes after the simulation period. Two severity levels were considered in our experiment, a loose severity $S_{1}=\left(T_{1}=4,T_{2}=6\right)$ in Figure 11a and a coarse severity $S_{2}=\left(T_{1}=2,T_{2}=5\right)$ in Figure 11b. From the figures, it can be seen that LIBP performs better for both severity levels as it was the only protocol that had the majority of its nodes in the ‘susceptible’ state after the simulation ended. On average, the CTP protocol also showed some stability as most of its nodes remained in the ‘attacked’ state for both severity levels. For both severity levels, we observed that using the RPL-ETX protocol, nodes were in the ‘removed’ state after the simulation, while RPL-0 moved nodes from the ‘attacked’ to the ‘removed’ state only under coarser severity. This means for the same operation time, a network running the LIBP protocol would have its nodes mostly fully charged. Comparatively, for a CTP network, the nodes would be averagely charged while nodes in a RPL operated network would have their batteries fully depleted. This is in agreement with previous works [3,4,38], which revealed the energy frugality of the LIBP protocol. In this context, the sink node is not considered as one of the nodes as it is assumed that it is connected to an external power source and hence does not run on batteries like the others.

#### 5.4.4. Summary

The results presented above reveal that the LIBP protocol leads to more nodes remaining in the susceptible state compared to other protocols on different performance profiles. This shows that it is more suitable for sensitive applications than RPL and CTP. RPL and CTP are cost-aware (using ETX metric) protocols and are more suitable for non-sensitive applications. These results reveal that a hybrid network engineering model that can combine interference-aware routing using LIBP and cost-aware routing using RPL or CTP might be an alternative to parameters adjustment and can provide a good trade-off between dependability in terms of safety and network overheads.

### 5.5. The Impact of INMP on the CPS Dependability

Using Cooja, we extended the multi-sink protocol in [4] to add migration capabilities to the nodes and assessed the impact of this on the CPS’ dependability. Using a novel protocol referred to as “INMP”, we conducted a number of experiments to evaluate the impact on CPS safety in terms of energy consumption and nodal interference which can be translated into epidemic states.

#### 5.5.1. Impact of Clustering on Energy Consumption

A first set of experiments was conducted to evaluate the energy consumption of a single IoT network compared to a partitioned network using clustering. The key performance parameter considered was the energy consumed by the sink node. Being the collection point of all data collected by other nodes, the sink node is one of the most energy-consuming nodes in an IoT network. The results in Table 3 revealed that the maximum energies consumed by the sink nodes when the network was partitioned into 2, 3, 4, and 5 clusters were respectively 5.86%, 6.0%, 5.71%, and 5.84% of the total energy consumed by the entire IoT network while on the average, it consumed 5.07%, 4.38%, 3.92%, and 4.23% of the total IoT network’s energy. A higher sink energy consumption of 6.60% was recorded when the IoT network was designed with a single sink. This revealed the relevance of using network partitioning as an efficient network reconfiguration strategy to lower the energy consumption of the CPS-IoT subsystem.

#### 5.5.2. Impact of Node Migration

A second set of experiments was conducted to evaluate the benefits of moving nodes from one cluster to another in a multi-sink clustered configuration. An initial cluster configuration Configuration 1: (10, 21, and 17) was designed with each of the three clusters containing 10, 21, and 17 nodes respectively.

This reflected a network with high variance in terms of number of nodes in the respective clusters. Thereafter, the initial configuration was adjusted by moving 5 nodes from the cluster 2 to cluster 1, thus leading to the second clustering: Configuration 2: (15, 16, and 17). A third and balanced configuration, Configuration 3: (15, 15, and 15) with equal number of nodes in each cluster was then built from Configuration 2 by moving a node from cluster 3 to cluster 1. The impacts of node migration are reported on Table 4 and Table 5 in terms of energy consumption and nodes’ interference respectively. As reported in Table 4, the energy consumption resulting from these network re-configurations reveals a balance in energy consumption due to node migration. A reduction in energy consumption variances of sink nodes was observed as we moved from an unbalanced network (Configuration 1, maximum energy variance of 0.4657) to a more balanced network (Configuration 3, maximum energy variance of 0.0009). Similarly, Table 5 reveals that besides energy conservation, the CPS-IoT reconfiguration using node migration also leads to a balanced profile in terms of interference. This in turn translates to a higher probability of prolonging the lifetime of the CPS-IoT.

To test the scalability of our solution, we setup a bigger IoT network consisting of 86 nodes. We partitioned this into two clustering configurations—an unbalanced Configuration4: (49, 37) and a balanced Configuration5: (43, 43). Table 6 and Table 7 further confirm previous results and reveal that node migration between clusters can increase the safety of the CPS-IoT by reducing energy consumption and can lead to a more balanced CPS-IoT in terms of an interference profile.

## 6. Conclusions

This paper addressed the issue of the dependability of CPS by using mathematical epidemiology to model the CPS-IoT monitoring process and proposed a new reconfiguration protocol that uses network partitioning through clustering and nodes migration between clusters to restore the safety of CPS-IoT subsystems. A CPS-IoT monitoring model was proposed as a key component of a CPS orchestration system that uses a closed loop to affect inbound surveillance of the physical space and outbound actuation (based on epidemic curves visualised and analysed in the cyber space of the CPS). The model employs state transitions to identify the combination of parameters best suited for both sensitive and non-sensitive applications. Numerical analysis of the proposed model revealed that: (i) The transmission between diffusion sets could indeed be used to quantify energy usage in the CPS-IoT subsystem by showing that an increase in interference could cause nodes to move across states. (ii) By adjusting the migration rates based on the networking needs, high availability and/or fault tolerance/resilience, could be achieved. (iii) With predictable migration time, the proposed model could mitigate the destructive effect of interference on the network, especially when deployed in an autonomous mode. Finally, the experimental results of the proposed interference-aware clustering protocol with node migration revealed the relevance of the partitioning of the network in terms of energy consumption and interference as well as the beneficial impact of balancing nodes across the CPS-IoT network.

Amending CTPs algorithms to track the diffusion sets occupancy in order to move the path selection towards a given application specific behaviour is an avenue for future work. Achieving Quality of Service (QoS) in the CPS-IoT subsystem through service differentiation as done in [43] can also be addressed as extensions to this work. Finally, network reconfiguration through parameters optimisation could also be another possible direction for further research work. The extension of the work done in [44,45] with the techniques proposed in this paper when viewing teams of drones as cyber physical systems is another avenue for future work.

## Figures and Tables

**Table 1:** Safety perspective to CPS dependability.

Safety	S-to-A	A-to-R	Case
	Availability	Reliability	
Low dependability			
Low availability, low reliability	1	0	1
Average dependability			
High availability, low reliability	0	0	2
Worse dependability			
Low availability, high reliability	1	1	3
High dependability			
High availability, high reliability	0	1	4

**Table 2:** Diffusion model’s parameters.

Parameter	Description	Value
N	Total number of nodes	100
m	Number of diffusion sets	4
$S_{i}^{0}$	Initial number of susceptible nodes in set *i*	$S_{1}^{0}=95$, $S_{2}^{0}=70$, $S_{3}^{0}=60$, $S_{4}^{0}=50$
$A_{i}^{0}$	Initial number of attacked nodes in set *i*	$A_{1}^{0}=5$, $A_{2}^{0}=30$, $A_{3}^{0}=40$, $A_{4}^{0}=50$
$R_{i}^{0}$	Initial number of removed nodes in set *i*	$R_{1}^{0}=0$, $R_{2}^{0}=0$, $R_{3}^{0}=0$, $R_{4}^{0}=0$
$\lambda_{ij}$	Transmission rate from $S_{i}$ to $A_{j}$	$\lambda_{12}=0.04$, $\lambda_{21}=0.03$, $\lambda_{ij}=0$, with i>2 or j>2
$\rho_{ij}$	Transmission rate from $A_{i}$ to $R_{j}$	$\rho_{12}=0.01=\rho_{21}$, $\rho_{ij}=0$, with i>2 or j>2
$b_{i}$	Migration rate from $S_{i}$ to $A_{i}$	gamma random distribution
$c_{i}$	Migration rate from $A_{i}$ to *i*	$c_{1}$, $c_{2}$, $c_{3}$, $c_{4}=0.02$
$\beta_{i}$	Susceptibility of a node in diffusion set *i*	$\beta_{1}$, $\beta_{2}$, $\beta_{3}$, $\beta_{4}=0.11$
$\gamma_{i}$	Infectiousness of a node in diffusion set *i*	$\gamma_{1}$, $\gamma_{2}$, $\gamma_{3}$, $\gamma_{4}=4.0$

**Table 3:** Impact of clustering on energy consumption.

	5 Clusters	4 Clusters	3 Clusters	2 Clusters	1 Cluster
Sink1:	4.55%	5.71%	3.18%	5.86%	6.60%
Sink2:	5.84%	3.86%	6.00%	4.27%	none
Sink3:	3.48%	3.40%	3.96%	none	none
Sink4:	3.35%	3.28%	none	none	none
Sink5:	3.93%	none	none	none	none
Average:	4.23%	3.92%	4.38%	5.07%	6.6%

**Table 4:** Node migration: Energy consumption.

	Configuration 1		Configuration 2		Configuration 3	
Energy	Highest	Average	Highest	Average	Highest	Average
Sink1	4.18%	2.09%	4.77%	2.51%	4.77%	2.58%
Sink2	5.54%	2.89%	4.83%	2.66%	4.83%	2.66%
Sink3	4.96%	2.44%	4.85%	2.71%	4.79%	2.61%
Variance	0.4657	0.1608	0.0017	0.0108	0.0009	0.0016

**Table 5:** Node migration: Node interference.

	Configuration 1			Configuration 2			Configuration 3		
	0	1	2	0	1	2	0	1	2
Sink1	4	4	2	6	6	3	7	6	3
Sink2	10	7	4	7	6	3	7	6	3
Sink3	7	7	3	7	7	3	6	7	3
Variance	4.33	3	1	0.33	0.33	0	0.33	0.33	0

**Table 6:** Node migration: Energy consumption (86-nodes).

	Configuration 4		Configuration 5	
	Highest	Average	Highest	Average
Sink4	6.6%	3.9%	6.48%	3.88%
Sink5	6.3%	3.79%	6.55%	3.92%
Variance	0.045.	0.0162	0.00245	0.0008

**Table 7:** Node migration: Node interference (86-nodes).

	Configuration 4				Configuration 5			
	0	1	2	3	0	1	2	3
Sink4	25	12	8	4	22	10	7	4
Sink5	19	9	6	3	20	11	8	4
Variance	18	4.5	2	0.5	1.414	0.707	0.5	0

## Data Availability

Not applicable.
